# Effectiveness of sodium thiosulfate in acquired reactive perforating collagenosis: A case report

**DOI:** 10.1097/MD.0000000000046992

**Published:** 2026-01-09

**Authors:** Bocheng Zhu, Keqing Zhu, Liyu Pan, Xiaoyang Guan, Xiaofeng Zhu, Chenxia Wu, Shengxian Shen

**Affiliations:** a Department of Nephrology, No. 903 Hospital of PLA Joint Logistic Support Force, Hangzhou, China; b Department of Dermatology, No. 903 Hospital of PLA Joint Logistic Support Force, Hangzhou, China.

**Keywords:** Th2-type inflammation, acquired reactive perforating collagenosis, case report, renal dialysis, sodium thiosulfate

## Abstract

**Rationale:**

Acquired reactive perforating collagenosis (ARPC) is a challenging dermatological complication in end-stage renal disease (ESRD) patients on hemodialysis, characterized by transepidermal elimination of degenerated collagen and intense pruritus. Treatment options are limited and lack consensus. Based on its established safety profile in ESRD and pathophysiological rationale, we evaluated sodium thiosulfate (STS) as a novel therapeutic option for ARPC.

**Patient concerns:**

A 70-year-old male with ESRD on hemodialysis and type 2 diabetes presented with widespread pruritic papules consistent with ARPC.

**Diagnoses:**

The diagnosis of ARPC was confirmed histologically by Masson trichrome staining.

**Interventions:**

After unsatisfactory responses to topical steroids and antihistamines, intravenous STS (3.2 g after hemodialysis, 3 times weekly) was initiated.

**Outcomes:**

The patient showed marked clinical improvement in pruritus and skin lesions within 1 month, with a corresponding decrease in eosinophil count and IgE levels. No adverse effects, such as metabolic acidosis or gastrointestinal symptoms, were observed.

**Lessons:**

STS may be an effective and well-tolerated treatment for ARPC in hemodialysis patients. The observed immunomodulatory changes, though possibly related to symptom resolution, support further investigation into its mechanism of action.

## 1. Introduction

Acquired Reactive Perforating Collagenosis (ARPC) is a type of perforating dermatosis.^[1]^ The affected population of ARPC consists of adults aged 18 years and older, characterized by umbilicated papules or nodules on the skin surface with central adherent keratotic plugs, and histopathology showing basophilic collagenous tissue expelled through the epidermis, with clinical manifestations of severe skin itching.^[2]^ The pathogenesis of ARPC is unclear, but diseases such as diabetes, end-stage renal disease (ESRD) requiring maintenance hemodialysis, and hyperthyroidism have been reported to be closely associated with its onset.^[3,4]^ In this case report, we present a case of chronic renal failure requiring maintenance hemodialysis combined with ARPC, which was also complicated by type 2 diabetes and hypertension. The skin lesions of this case were effectively controlled through treatment with intravenous sodium thiosulfate (STS).

## 2. Case report

**Figure 1.**
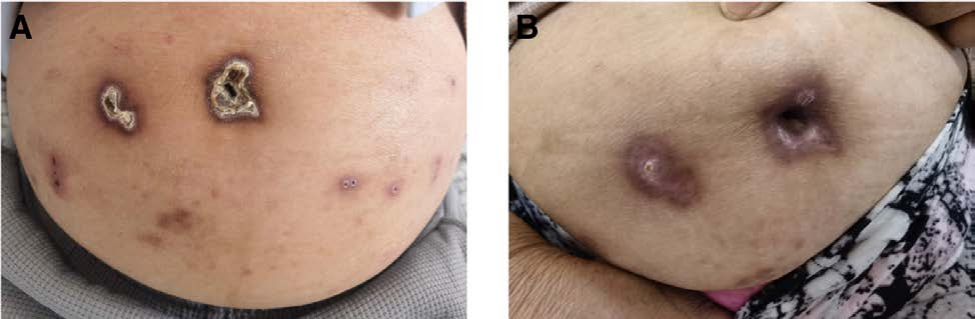
(A) Before treatment, the patient had multiple umbilicated papules and nodules with central keratotic crusts on the abdomen. (B) After treatment with sodium thiosulfate, these lesions on the abdomen significantly regressed, leaving behind residual hyperpigmentation.

The patient is a 70-year-old male who was admitted to the hospital with a history of maintenance hemodialysis for 10 months and multiple rashes on the trunk and extremities for over 1 year. The timeline of key clinical events and interventions is summarized in Table 1. Nine years ago, the patient was hospitalized in another facility where elevated blood creatinine (155 μmol/L) was discovered, leading to a diagnosis of “type 2 diabetes, stage IV diabetic nephropathy, and stage 3 chronic kidney disease.” After symptomatic treatment, the patient improved and was discharged, with regular outpatient follow-up. One year ago, the patient was hospitalized due to progressive fatigue, poor appetite, chest tightness, shortness of breath, and lower extremity edema, and began routine hemodialysis treatment in early 2024 (three times a week), consisting of 2 hemodialysis sessions and 1 hemodiafiltration session per week. One year ago, the patient developed scattered brown rashes on the extremities ranging from millet to soybean size, accompanied by itching; Upon admission, the rashes had gradually increased, involving the chest, abdomen, and back. Due to skin itching, there were prominent scratch marks on the trunk and extremities, with some areas presenting as ulcerative plaques (Fig. 1A).

**Table 1:** Timeline of key clinical events and interventions.

Event/intervention	Time relative to presentation	Details
Diagnosis of diabetic nephropathy and CKD	9 yr prior	Serum creatinine 155 μmol/L (stage 3 CKD)
Onset of skin rash and pruritus	1 yr prior	Scattered brown rashes on extremities, ranging in size, with intense itching (NRS = 10)
Initiation of maintenance hemodialysis	10 mo prior	Routine hemodialysis began (3 times/wk) due to ESRD
Prior topical treatment	~1 yr prior	Topical steroid ointments and moisturizers (unsatisfactory effect)
Prior systemic treatment	~1 yr prior	Oral antihistamines (led to mental fatigue and somnolence)
Initiation of sodium thiosulfate	At admission	3.2 g IV, 3 times/wk after hemodialysis
Clinical follow-up	1 mo after sodium thiosulfate	Significant improvement in pruritus (NRS = 4); resolution of skin lesions

CKD = chronic kidney disease, ESRD = end-stage renal disease.

**Figure 2.**
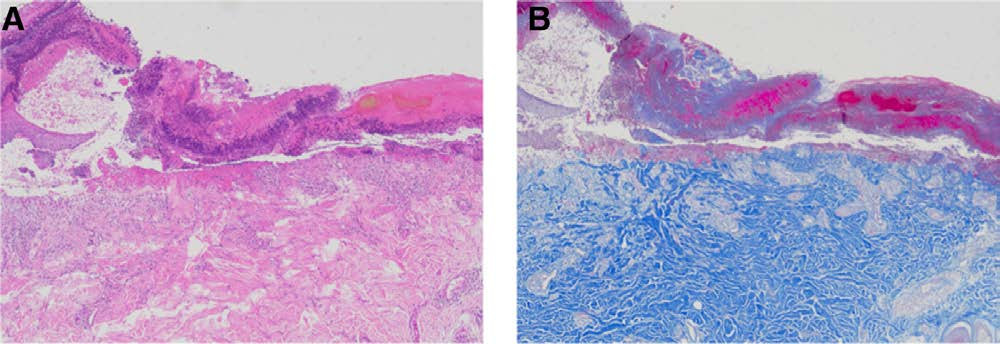
(A) Lesional skin biopsy showing ulcerative changes in the epidermis with necrotic tissue fragments, superficial dermal collagen deposition, degeneration of reticular collagen fibers, and sparse chronic inflammatory cell infiltration around blood vessels. (B) Masson trichrome staining showing elimination of fibrous tissue through the dermis into the epidermis. (A) × 100. (B) × 100.

Upon admission, laboratory tests revealed hemoglobin (78 g/L), eosinophils (1.66 × 10⁹/L), C-reactive protein (17.69 mg/L), erythrocyte sedimentation rate (101 mm/H), fasting blood glucose (5.96 mmol/L), glycated hemoglobin (6.2%), urea (26.04 mmol/L), creatinine (483μmol/L), and immunoglobulin E (619.7 KIU/L, normal reference range: ≤158 KIU/L). Thyroid function, screening for acquired immunodeficiency syndrome (AIDS), rapid plasma reagin test, serological markers for hepatitis B virus, hepatitis C virus antibodies, and antinuclear antibodies were all normal. The numerical rating scale for itching showed a score of 10. A dermatology consultation was conducted, revealing umbilicated lesions and a positive Koebner phenomenon. Histopathological examination of a skin biopsy from the lesions showed ulcerative changes in the epidermis with necrotic tissue fragments, superficial dermal collagen deposition, degeneration of reticular collagen fibers, and sparse chronic inflammatory cell infiltration around blood vessels (Fig. 2A). Masson trichrome staining confirmed that degenerated collagen penetrated from the dermis to the epidermis, establishing the diagnosis of ARPC (Fig. 2B).

We administered hemodialysis 3 times a week, subcutaneous insulin injections to control blood glucose, oral calcium channel blockers for blood pressure management, and supplements of folic acid and vitamin B complex. Due to the previous unsatisfactory effects of topical steroid ointments and oral antihistamines, the patient received intravenous sodium thiosulfate (3.2 g/administration) 3 times a week after hemodialysis. After 1 month, the patient’s skin itching significantly improved, and the skin lesions resolved (Fig. 1B). Follow-up tests revealed hemoglobin (88 g/L), eosinophils (1.33 × 10⁹/L), C-reactive protein (7.14 mg/L), erythrocyte sedimentation rate (55 mm/H), immunoglobulin E (235 KIU/L, normal reference range: ≤158 KIU/L), and an numerical rating scale score of 4.

To evaluate potential confounding factors for pruritus, we also monitored parameters related to mineral metabolism and dialysis adequacy. The patient’s serum phosphate (1.78 mmol/L at baseline vs 1.82 mmol/L at 1-month) and intact parathyroid hormone level (285 pg/mL vs 302 pg/mL) remained stable and within the target range for ESRD. Furthermore, dialysis adequacy, measured by single-pool Kt/V, was maintained at 1.45. The stability of these conventional risk factors supports the conclusion that the clinical improvement was specifically associated with sodium thiosulfate therapy.

The patient’s tolerance to sodium thiosulfate was excellent. He reported no gastrointestinal side effects, such as nausea or vomiting, throughout the 1-month treatment period. Metabolic acidosis was actively monitored by routinely measuring serum bicarbonate levels before and during therapy. The patient’s serum bicarbonate levels remained stable within the normal range for a hemodialysis patient, indicating no significant induction or worsening of metabolic acidosis.

## 3. Discussion

ARPC has been reported in only a small number of patients, and there is no unified statistical data on its exact incidence in hemodialysis patients. A 1982 report from North America indicated that ARPC occurred in 4.5% to 10% of hemodialysis patients.^[5–7]^ In a dialysis center in the UK, the incidence of ARPC in the dialysis population was 11%, with an incidence of 8.3% in the hemodialysis group and as high as 16.67% in the peritoneal dialysis group.^[8]^ However, it should be noted that these data come from small sample studies under specific conditions and may not represent the actual situation of all dialysis patients. The etiology and pathogenesis of ARPC remain unclear. Current literature reports that ARPC is associated with endocrine diseases, kidney diseases, cardiovascular diseases, gastrointestinal diseases, vascular diseases, tumors, and autoimmune diseases.^[4]^ Among these, ESRD and diabetes are closely related to the onset of ARPC.^[8]^ Skin itching is a common clinical symptom in hemodialysis patients,^[9]^ and scratching due to pruritus leads to superficial skin trauma, microvascular changes in diabetic nephropathy, and skin calcium deposition in ESRD, all contributing to the development of ARPC.^[3,10]^ Some studies have also reported that significant azotemia in ESRD patients undergoing hemodialysis causes changes in the chemical composition of the dermal layer of the skin, which may trigger pruritus, leading to collagen degeneration in the dermis and expulsion through the skin, forming hyperkeratotic papules and other skin lesions, which is a typical feature of ARPC.^[3]^ The onset of ARPC generally shows no gender difference, though some literature reviews have found a higher incidence in males (ratio of 1.5:1).^[3]^ The main manifestations of ARPC are pruritus and hyperkeratotic papules, which are umbilicated or volcano-like, often with central keratotic plugs. The diameter of the papules is typically 0.5 to 2.0 cm, and some may fuse and enlarge. These papules are primarily distributed on the trunk and extremities, particularly on the extensor surfaces, especially the buttocks, and may also appear on the cheeks and forehead, often with Koebner phenomenon, which has been confirmed that 83.3% of 30 ARPC cases had pruritus, and 31.8% had Koebner phenomenon.^[11]^

In 1994, the diagnostic criteria for ARPC was proposed, which include the following 3 conditions^[2]^: histopathology indicating necrotic basophilic collagen tissue expelled into an epidermal cup-shaped depression; clinical presentation of umbilicated papules or nodules with central adherent keratotic plugs; and the appearance of skin lesions after the age of 18. Histopathological findings include^[12]^: hematoxylin and eosin staining revealing cup-shaped epidermal depression with incomplete keratin, cell fragments, and basophilic collagen fibers, with fragmented and coiled collagen fibers vertically penetrating the epidermis; the superficial dermis shows infiltration of numerous neutrophils and a few lymphocytes; Masson trichrome staining reveals blue-stained, broken, fragmented, and degenerated collagen fibers penetrating the epidermis^[13]^; elastic van Gieson staining shows red-colored perforated collagen.^[14]^

The diagnosis of ARPC in this case was confirmed histopathologically, but it is important to distinguish it from other perforating dermatoses, notably Kyrle disease, perforating folliculitis, and elastosis perforans serpiginosa.^[4]^ Although these conditions share the common histological finding of transepidermal elimination, the nature of the eliminated material and the associated clinical presentation differ. Kyrle disease is characterized by the elimination of abnormal keratin, not collagen, and lesions are often larger and more hyperkeratotic, frequently associated with underlying renal disease or diabetes. Perforating folliculitis primarily involves the hair follicle, with perforating tracts containing basophilic debris and hair fragments, and clinically presents as follicular papules or pustules. Elastosis perforans serpiginosa involves the elimination of abnormal elastic fibers, which can be confirmed with elastic fiber stains (e.g., Verhoeff-Van Gieson), and typically presents with serpiginous or arcuate clusters of papules in young individuals, often associated with genetic disorders. In our patient, the histopathological findings were definitive for ARPC: Hematoxylin and eosin staining showed transepidermal elimination of basophilic degenerated collagen fibers, which was further confirmed by Masson trichrome staining that highlighted the blue-stained collagen bundles. The absence of follicular centrality or abnormal elastic fibers, combined with the clinical presentation of widespread umbilicated papules in an elderly patient with ESRD and diabetes, strongly favored the diagnosis of ARPC over its differentials.

Currently, there is no standardized treatment protocol for ARPC. The main treatment strategies include topical medications, phototherapy, and systemic drug administration. Some patients exhibit a self-limiting course, while others require aggressive intervention. In our patient, the failure of conventional therapies (topical steroids, moisturizers, and antihistamines) necessitated the use of a systemic agent. The decision to employ sodium thiosulfate was based on a confluence of factors. It was selected over other systemic options due to its favorable safety profile in the hemodialysis population, a pathophysiological rationale aligned with the patient’s comorbidities, and our positive institutional experience with its use in calciphylaxis. Unlike thalidomide, which carries significant risks of neuropathy,^[15]^ or novel biologics such as dupilumab^[16,17]^ and baricitinib,^[18]^ which have limited evidence, high cost, and potential immunosuppressive risks, sodium thiosulfate’s safety in dialysis patients is well-established.^[19]^ The potential benefit observed in our case may be explained by several hypothesized, though still theoretical, mechanisms of action. Its hypothesized dual mechanisms of action – strong antioxidant and anti-inflammatory properties (by scavenging reactive oxygen species and producing hydrogen sulfide),^[20–22]^ coupled with calcium chelating properties that may modulate calcium-phosphate metabolism^[19]^ – may offered a plausible approach to target the underlying microvascular, inflammatory, and potential calcific processes in ARPC, particularly in a patient with diabetes and ESRD. Furthermore, the analogous efficacy observed in calciphylaxis, a condition sharing a similar patient demographic and features of severe skin inflammation, provided a compelling clinical precedent.^[18,23]^

Furthermore, while the observed decrease in eosinophil count and IgE levels following sodium thiosulfate treatment is an interesting finding, its interpretation is limited by the observational nature of this case report. It remains uncertain whether this represents a direct immunomodulatory effect of the drug or an indirect consequence of the resolution of skin inflammation and pruritus, leading to a reduction in systemic inflammatory markers. This important distinction should be explored in future controlled studies.

## 4. Conclusion

To our knowledge, this is the first report to describe the successful and targeted use of intravenous sodium thiosulfate for the treatment of ARPC, providing details on its dosing regimen and clinical efficacy. This case highlights the efficacy of sodium thiosulfate in treating ARPC and suggests its consideration in ARPC cases resistant to conventional treatments. The effects of sodium thiosulfate treatment on the significant decrease in the patient’s eosinophil count and IgE levels, as well as the underlying mechanisms, warrant further validation and exploration.

## Acknowledgments

We thank the patient for allowing the publication of this case report.

## Author contributions

**Conceptualization:** Bocheng Zhu.

**Data curation:** Keqing Zhu, Shengxian Shen.

**Investigation:** Xiaoyang Guan.

**Supervision:** Xiaofeng Zhu.

**Validation:** Liyu Pan, Chenxia Wu.

**Writing – original draft:** Bocheng Zhu.

**Writing – review & editing:** Shengxian Shen.
